# ‘Kissing’ squamous cell carcinoma occurring simultaneously in the upper and lower eyelids

**DOI:** 10.1093/jscr/rjag926

**Published:** 2026-10-14

**Authors:** John T LiVecchi, Firoozeh Raygan, Yujia Zhou, David T Y Yang, Elizabeth S Yang, Anny M S Cheng

**Affiliations:** Department of Ophthalmology, University of Florida, 6201 W. Newberry Road, Gainesville, FL 32605, United States; Department of Ophthalmology, University of Florida, 6201 W. Newberry Road, Gainesville, FL 32605, United States; Department of Ophthalmology, University of Florida, 6201 W. Newberry Road, Gainesville, FL 32605, United States; Northeast Ohio Medical University, College of Medicine, 4209 State Route 44, Rootstown, OH 44272, United States; New York University, College of Arts and Sciences, 32 Waverly Pl, New York, NY 10003, United States; Department of Ophthalmology, Alaska Native Medical Center, 3976 University Lake Dr, Anchorage, AK 99508, United States

**Keywords:** eyelid reconstruction, Mohs surgery, squamous cell carcinoma, supraclavicular full-thickness skin graft, upper and lower eyelids

## Abstract

Eyelid squamous cell carcinoma (SCC) typically arises from eyelid skin; however, simultaneous involvement of both the upper and lower eyelids has not been previously reported. A 74-year-old Caucasian man presented with two distinct right eyelid masses: a 2-cm ulcerated upper eyelid lesion and a 1.5-cm lower eyelid lesion with prominent telangiectatic vessels. Biopsy confirmed SCC, and Mohs surgery achieved complete tumor removal. The resulting anterior lamellar defects measured 3.5 × 1.2 cm in the upper eyelid and 2.5 × 0.9 cm in the lower eyelid. Reconstruction was performed using a 7 × 2 cm full-thickness skin graft harvested from the supraclavicular region. At 3 months, there was no evidence of recurrence, eyelid function remained normal, and the cosmetic outcome was satisfactory. This rare presentation highlights the reconstructive challenges of extensive dual-eyelid SCC and emphasizes meticulous surgical reconstruction and long-term surveillance for recurrence and potential complications.

## Introduction

Squamous cell carcinoma (SCC) is the second most common cutaneous malignancy that on the eyelid [1]. Eyelid SCCs usually originate from eyelid skin, predominantly affecting the lower and medial eyelids before the upper eyelids [1]. While other aggressive eyelid malignancies, such as sebaceous carcinoma, have been reported to simultaneously involve multiple periocular skin zones including both upper and lower eyelids [2], such dual-eyelid involvement by SCC has not been previously reported in the literature. This absence highlights a unique diagnostic and management challenge.

Herein, we present a case report of a patient undergoing Mohs micrographic surgery for SCC concurrently affecting both the upper and lower eyelids of the same eye.​ The extensive defect necessitated delicate reconstruction involving multiple grafts to restore critical eyelid functions, including mobility and tear film stability, in addition to achieving an acceptable cosmetic outcome. This case underscores the reconstructive encountered when SCC manifests in this rare, extensive presentation, demanding a multidisciplinary approach for optimal patient management.

## Case presentation

A 74-year-old Caucasian male, with a notable medical history including a remote diagnosis of basal cell carcinoma on his arms, was referred for evaluation of a right eye eyelid mass that had been present for ~1 year.​ Clinical examination revealed two distinct lesions: one on the upper eyelid measuring 2 cm in diameter, and another on the lower eyelid measuring 1.5 cm in diameter. Both masses appeared round and erythematous. The upper eyelid lesion exhibited ulceration, while the lower eyelid lesion of the same eye was characterized by prominent dilated telangiectatic blood vessels (Fig. 1a). A thorough systemic examination found no evidence of regional lymphadenopathy or any other abnormalities in the periorbital or facial areas. Ophthalmic assessment indicated a visual acuity of 20/25 in both eyes, with an intraocular pressure (IOP) of 15. The right eye presented with conjunctival injection, though the cornea remained clear, and posterior segment examination was unremarkable. The left eye’s slit lamp and fundus examinations also yielded unremarkable findings.

**Figure 1:**
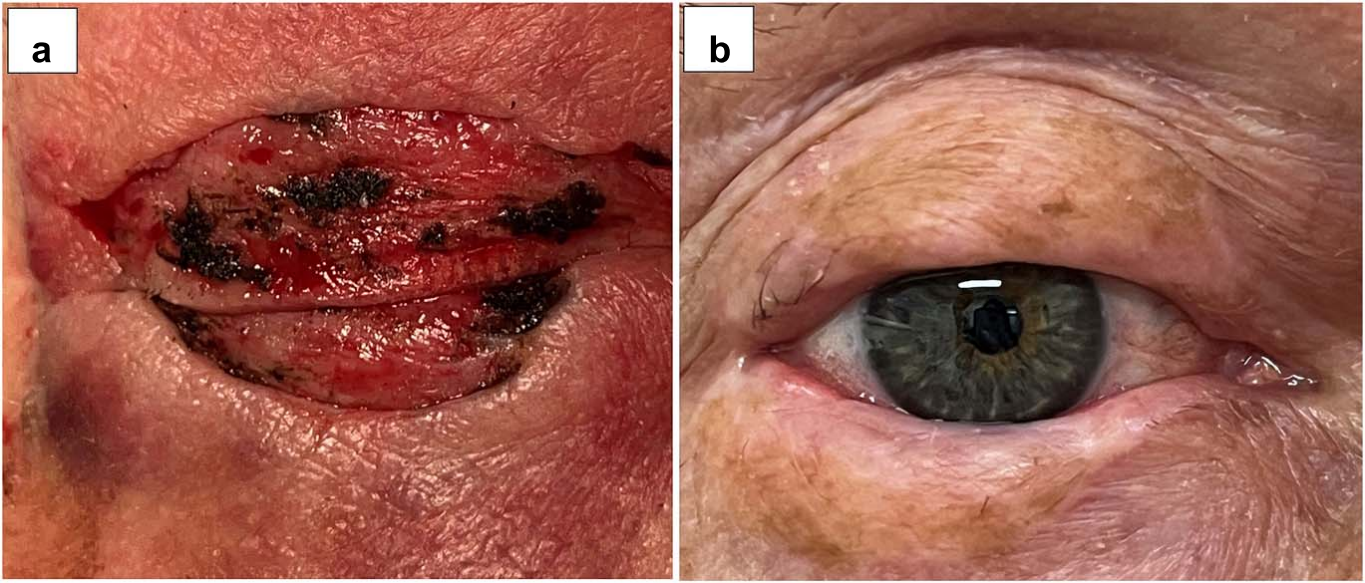
A SCC simultaneously involving both the upper and lower eyelids underwent Mohs surgery and subsequent eyelid defect reconstruction. The resulting upper and lower eyelid surgical defects after Mohns surgery (a), required reconstruction using full-thickness skin graft harvested from the supraclavicular area to restore eyelid function and achieve an acceptable cosmetic outcome (b).

Biopsy confirmed SCC without involvement of the palpebral conjunctiva.​ He subsequently underwent Mohs micrographic surgery for complete tumor removal. The surgical defects measured 3.5 × 1.2 cm in the upper eyelid and 2.5 × 0.9 cm in the lower eyelid. Both defects had clear margins confirmed during the procedure, and the posterior lamella was preserved. Reconstruction of both defects was performed using a 7 × 2 cm full-thickness skin graft harvested from the supraclavicular area. A #15 blade was used for the incision, and the resulting donor site was closed with 5-0 Vicryl sutures in the subcutaneous layer and skin. The harvested graft was then meticulously defatted to ensure optimal integration and viability. The prepared graft was trimmed to at 0.5 mm larger than the upper eyelid defect. The upper eyelid defect was closed using 6-0 fast-absorbing plain gut sutures placed in an interrupted fashion. This included critical lid margin closure sutures, which were carefully tied away from the ocular surface to prevent irritation. The same full-thickness skin graft harvesting, preparation, and suturing technique was then applied to reconstruct the right lower eyelid defect, aiming to restore both function and cosmetic appearance (Fig. 1b). Three months postoperatively, the patient exhibited no signs of recurrence. Normal eyelid levator function was observed, with no upper or lower eyelid ptosis, and the esthetic outcome was satisfactory.

## Discussion

The case of this elderly man typifies the age at presentation, clinical description, and histological findings of a SCC. However, this is the first case report of SCC simultaneously affecting both the upper and lower eyelids of the same eye, suggesting a potentially rare or previously unacknowledged clinical presentation.​ Such extensive involvement poses considerable challenges for both surgical removal and subsequent reconstruction.

Addressing SCC across both eyelids necessitates a multidisciplinary strategy. Due to the unique function and anatomy of the eyelids, Mohs micrographic surgery is recognized to be the gold standard for treating SCC [3]. A British study involving 34 patients with periocular SCC who underwent Mohs surgery highlighted not only its ability to preserve healthy tissue but also a significantly low rate of post-operative complications (2.9%) [4]. To prevent complications in reconstructive surgery, our primary goal is to eliminate tension, followed by the careful reconstruction of the resulting defect [5]. To achieve this, we intentionally harvested significantly oversized grafts for both the upper and lower eyelids. Poor initial reattachment of the eyelid’s delicate tissue layers and excessive vertical tension can lead to cicatricial changes. Therefore, during the reconstruction surgery, we meticulously ensured there was no laxity in the eyelids.

Various techniques have been proposed for reconstruction of the eyelid’s anterior lamella after tumor excision, including the transposition of skin flaps, full-thickness skin grafts, or combination of these two methods. We selected full thickness skin grafts, aligning with study that demonstrates excellent functional and esthetic outcomes with this method [6]. Given that the reconstruction involved both upper and lower eyelids, the supraclavicular area was chosen as a reliable donor site. This region provides skin with comparable color, thickness, and flexibility to the eyelids. This is particularly useful for harvesting a sufficiently large graft, especially when the contralateral eyelid and postauricular areas do not offer enough skin [7]. These attributes are crucial for achieving optimal esthetic and functional outcomes in the delicate eyelid region.

This unique case incorporated Mohs micrographic surgery for precise and complete tumor clearance, specialized oculoplastic surgeons for reconstruction, and potentially multiple subspecialities for adjuvant therapy. This multidisciplinary approach was important given SCC’s known aggressiveness and propensity for local tissue destruction, orbital invasion, perineural invasion, and lymphatic and hematogenous metastases [8]. This case presentation also highlights the importance of long-term follow-up care, particularly as there is currently no evidence of local or distance metastasis. Such surveillance is critical for early detection of any recurrence and for effectively managing potential late-onset complications arising from extensive surgery and reconstruction.

## Data Availability

The dataset supporting the conclusions of this article is included within the article (and its additional file).
